# Gas Transport Properties of Polybenzimidazole and Poly(Phenylene Oxide) Mixed Matrix Membranes Incorporated with PDA-Functionalised Titanate Nanotubes

**DOI:** 10.1186/s11671-016-1613-4

**Published:** 2017-01-03

**Authors:** V. Giel, M. Perchacz, J. Kredatusová, Z. Pientka

**Affiliations:** Institute of Macromolecular Chemistry, Academy of Sciences of the Czech Republic, Heyrovsky Sq. 2, 16206 Prague 6, Czech Republic

**Keywords:** Polybenzimidazole, Poly(phenylene oxide), Titanate nanotubes, Polydopamine, Mixed matrix membrane, Gas separation, Permeability, Selectivity, Sorption isotherms

## Abstract

Functionalised titanate nanotubes (TiNTs) were incorporated to poly(5,5-bisbenzimidazole-2,2-diyl-1,3-phenylene) (PBI) or poly(2,6-dimethyl-1,4-phenylene oxide) (PPO) for improving the interfacial compatibility between the polymer matrix and inorganic material and for altering the gas separation performance of the neat polymer membranes. Functionalisation consisted in oxidative polymerisation of dopamine-hydrochloride on the surface of non-functionalised TiNTs. Transmission electron microscopy (TEM) confirmed that a thin polydopamine (PDA) layer was created on the surface of TiNTs. 1.5, 3, 6, and 9 wt.% of PDA-functionalised TiNTs (PDA-TiNTs) were dispersed to each type of polymer matrix to create so-called mixed matrix membranes (MMMs). Infrared spectroscopy confirmed that –OH and –NH groups exist on the surface of PDA-TiNTs and that the nanotubes interact via H-bonding with PBI but not with PPO. The distribution of PDA-TiNTs in the MMMs was to some extent uniform as scanning electron microscope (SEM) studies showed. Beyond, PDA-TiNTs exhibit positive effect on gas transport properties, resulting in increased selectivities of MMMs. The addition of nanotubes caused a decrease in permeabilities but an increase in selectivities. It is shown that 9 wt.% of PDA-TiNTs in PBI gave a rise to CO_2_/N_2_ and CO_2_/CH_4_ selectivities of 112 and 63 %, respectively. In case of PPO-PDA-TiNT MMMs, CO_2_/N_2_ and CO_2_/CH_4_ selectivity increased about 25 and 17 %, respectively. Sorption measurement showed that the presence of PDA-TiNTs in PBI caused an increase in CO_2_ sorption, whereas the influence on other gases is less noticeable.

## Background

Nanomaterials have attracted considerable interest in many applications, including the field of membrane science [1–7]. In the last decades, numerous works have been published on the use of inorganic particles in various polymeric membrane structures and their functionalities [1, 8–13]. The goal of such so-called mixed matrix membranes (MMMs) is to achieve a system with more useful structural or functional properties unattainable by any of the constituent itself which may help to overcome the efficiency-productivity trade-off of neat polymer materials [14].

Thus far, a wide range of nanoparticles were used in MMMs, e.g. zeolites, metal organic frameworks (MOFs), mesoporous silicas, carbon molecular sieves, or carbon nanotubes (CNTs) [9, 15–17]. The choice of nanoparticles for the desired gas separation is of greatest significance, because major variables as gas adsorption or molecular sieving abilities of the nanoparticles may seriously affect the MMM performance. Beyond, uniformly dispersed nanoparticles in the polymer matrix as well as interfacial bonding notably influence gas transport properties [9, 16, 18].

Hitherto, CNTs and their potential for MMMs have been studied in great detail and seems to be a prospective filler for overcoming the efficiency-productivity trade-off of neat polymer membranes because of their high aspect ratio [1, 19]. Several polymeric materials have been tested to prepare MMMs as shown later in the text to alter their gas separation characteristics. For example, Wang et al. has embedded multi-walled nanotubes (MWNT) into PEG-based Pebax solution to separate CO_2_/CH_4_ and CO_2_/N_2_, respectively [20]. Another study of Pebax membranes was performed by Murali et al. [21]. It was shown that MWNT in the Pebax matrix enhances substantially the permeability of H_2_, O_2_, CO_2_, and N_2_. Khan et al. incorporated pristine MWNTs into PIM-1, which caused as well an increase in gas permeabilities [12]. Rajabi et al. reported on the addition of functionalised MWNT to polyvinylchloride (PVC) membranes which resulted in better gas separation performance, especially for CO_2_/CH_4_ [22]. Studies by Kim and his group demonstrated that the permeabilities of O_2_, N_2_, and CH_4_ increased proportionally to the amount of open-ended CNTs in the polymer matrix [23]. Li and his co-workers prepared MMMs from Matrimid and a combination of CNT and graphene oxide [24]. It was found that the MMMs with CNTs and graphene oxide had better gas separation performance than those with only one of these components, showing excellent separation properties. Cong et al. added single-walled carbon nanotubes (SWNT) and MWNT to brominated poly(2,6-dimethyl-1,4-phenylene oxide) (PPO) for CO_2_/N_2_ separation [25]. They observed that the MMMs had an increased CO_2_ permeability, while the CO_2_/N_2_ selectivity remained the same. In addition, permeabilities increased with the content of CNT.

However, there are several drawbacks regarding the use of CNTs in MMMs [1, 19]: (1) extreme amount of energy consumption because of high process temperatures (1000–3700 °C); (2) expensive fabrication owing to lasers and inert atmosphere; and (3) additional purification steps necessary due to by-products. With respect to these major issues, the utilisation of new fillers in MMMs is desirable.

Hence, this work focuses on titanate nanotubes (TiNTs), a relatively new class of nanotubes made of non-carbon material. These nanotubes possess similar morphology as CNTs, but the synthesis is carried out at far lower temperatures and at low cost [26, 27]. Moreover, variations of TiNT compositions and structures may be practically unlimited and the possibility of functionalisation is a useful way of treatment that may improve adhesion to polymer matrices. To date, most research about TiNTs has been directed towards the efficient synthesis and functionalisation of this filler as well on investigation of its unique morphology and physico-chemical properties [27–29]. However, almost all underlying studies about nanotubes for use in MMMs are connected to CNTs. Therefore, this study describes membranes altered by functionalised TiNTs and continues our earlier work in which and non-modified TiNTs were added to poly(5,5-bisbenzimidazole-2,2-diyl-1,3-phenylene) (PBI) or PPO [30]. Although various researches have been carried out on those polymers with diverse inorganic fillers, e.g. ZIF-8 [31], SBA-15 [32], silica nano particles [33, 34], there is so far no academic literature available on using functionalised TiNTs combined with PBI or PPO as membrane materials for gas separation, to the best of our knowledge. Therefore, the aim was to investigate the effect of TiNT on the gas transport properties of PPO and PBI membranes. It was found that the incorporation of non-modified TiNTs to PPO formed unselective voids, because the obtained ideal selectivities remained constant while permeabilities of all investigated gases increased. In case of PBI, the compatibility of non-modified TiNTs and the PBI matrix was enhanced because of the Ti–O groups present in TiNT which could interact with the N–H bonds present in PBI. These interactions increased the effective path of the penetrants, resulting in lower permeabilities and higher ideal selectivities.

Therefore, in continuation of this study [30], we attempted to functionalise the TiNTs in order to improve the adhesion between the filler and the polymer matrix, targeting to diminish the unselective voids and thus improving the membrane separation characteristics.

For the functionalisation was utilised polydopamine (PDA), because the formation of PDA layers onto nanotubes emerged as very efficient and facile [35–38]. Besides, PDA appears as a promising adhesive for subsequent surface-mediated reactions which allows tailoring properties according the used source materials [39, 40].

In the present work, the feasibility of the formation of MMMs based on PBI or PPO, respectively, and various amounts of PDA-TiNTs was studied. The as-prepared MMMs were characterised for their morphology, physico-chemical properties, and gas separation performance, aiming to investigate the effect of PDA-TiNT content on PBI or PPO membranes.

## Methods

### Materials

The PPO powder was purchased from Spolana Neratovice (Czech Republic). For dissolving PPO chloroform (Lachner, Czech Republic) was used as received.

Poly(5,5-bisbenzimidazole-2,2-diyl-1,3-phenylene) (PBI) was supplied by Hoechst Celanese and used as received as a 10 wt.% *N*,*N*-dimethylacetamide (DMAc) solution with a lithium chloride content of ~2 wt.%.

TiO_2_ powder (rutile modification), dopamine-hydrochloride, and tris(hydroxymethyl)aminomethane (TRIS) buffer (Sigma 7-9™, 99 %) were purchased from Sigma Aldrich, Germany.

### Nanotubes Synthesis and Functionalisation

TiNTs were synthesised by hydrothermal treatment. The details of the synthesis method of TiNTs are described elsewhere [30]. A thin PDA layer was created onto the surface of TiNTs by oxidative polymerisation of dopamine-hydrochloride. This procedure involves mixing of 800 ml of TiNT suspension (2 mg/ml) with 1.6 g of dopamine-hydrochloride dissolved in 5 g of TRIS buffer adjusted to pH = 8.5. The PDA coating on TiNTs was carried out for 3.5 h at 25 °C. After coating, the pH was adjusted to 6.4 using 35 wt.% HCl. The suspension was purified using dialysis tubing cellulose membrane and the resulting PDA-functionalised TiNTs (PDA-TiNTs) were isolated using freeze drying.

### Preparation of Mixed Matrix Membranes

Two series of MMMs were prepared: PPO-PDA-TiNT MMMs and PBI-PDA-TiNT MMMs. For the preparation of PPO-PDA-TiNT MMMs, PPO was dissolved in chloroform to obtain a 5 wt.% casting solution and stirred for 24 h. The PDA-TiNT was then added to the polymer solution and stirred with a magnetic stirrer for 24 h. The content of PDA-TiNT in the membranes was 1.5, 3, 6, and 9 wt.%, respectively. In case of PBI-PDA-TiNT MMMs, the PBI solution was diluted with DMAc to obtain as well a 5 wt.% polymer solution. The same amounts of PDA-TiNT were added as well to the polymer solutions of PBI. The details of the membrane preparation have been described in the previous work [30]. The resulting membranes had a thickness between 20 and 60 μm.

### Characterisation and Measurements

The size and morphology of TiNTs were analysed by using a transmission electron microscope (TEM) Tecnai G Spirit (FEI, 120 kV).

The specific surface area (*S*_BET_) of the TiNT and PDA-TiNT samples was measured by a gas adsorption technique on a Gemini VII 2390 (Micromeritics Instruments Corp., Norcross, USA) with nitrogen as the sorbate. The surface area was calculated from the Brunauer-Emmett-Teller (BET) adsorption/desorption isotherm using the Gemini software. It characterises materials in the region of micropores (<2 nm) [41].Calculations were done with a sample density *ρ* = 1.3 g/ml.

The cross section and surface morphology of the membranes were observed using a Quanta 200 FEG scanning electron microscope.

The thermal behaviour of the neat polymers and MMMs were studied on DSC Perkin Elmer 8500. The heating was performed in two steps. The first step was the continuous heating at 100 °C for 1 h in order to remove the volatiles from the polymer matrix. The second step was the increase of the temperature from 0 to 500 °C at the rate of 100 °C/min in a nitrogen purge (25 cm^3^/min).

Wide-angle X-ray scattering (WAXS) experiments were performed using a pinhole camera (modified Molecular Metrology System, Rigaku, Japan) attached to a micro-focused X-ray beam generator (Rigaku MicroMax 003) operating at 50 kV and 0.6 mA (30 W). The camera was equipped with removable and interchangeable Imaging Plate 23 × 25 cm (Fujifilm). Experimental setup covered the momentum transfer (*q*) range of 0.4–3.6 Å^−1^. While *q* = (4π/λ)sinθ, where *λ =* 1.54 Ǻ is the wavelength and 2θ is the scattering angle. Calibrations of the centre and sample-to-detector distance were made using Si powder. Samples were measured in transmission mode for 30 min.

The FTIR spectra of membranes were obtained using Spectrum 100 spectrometer (PerkinElmer, USA) equipped with a mercury–cadmium–telluride (MCT) detector in the wavelength range from 650 to 4000 cm^−1^ and universal attenuated total reflectance accessory (ATR) with a diamond prism. Spectral resolution was 4 cm^−1^ with 16 scans taken for each spectrum. The FTIR spectra of the nanotubes (TiNT, PDA-TiNT) and PDA were recorded on a Perkin Elmer Paragon 1000PC FTIR spectrometer using the reflective ATR technique Specac MKII Golden Gate Single Reflection ATR System with a diamond crystal. All spectra were measured in the wavenumber range 450–4400 cm^−1^ with a resolution of 4 cm^−1^ and with 32 scans.

Gas permeability through membranes was determined by the procedure described elsewhere [30]. The permeability *P* was determined from the increase of pressure Δ*p*_p_ per time Δ*t* and calculated via the following formula (Eq. 1) [42]:

1 $$ P=\frac{\varDelta {p}_{\mathrm{p}}}{\varDelta t}\cdot \frac{V_{\mathrm{p}}\cdot \kern0.5em l}{A\cdot {p}_{\mathrm{i}}}\cdot \frac{1}{\mathrm{R}T}, $$

where *l* is the membrane thickness, *A* the area, *T* the temperature, and R the gas constant. Permeabilities are reported in units of Barrer (1 Barrer = 1 × 10^−10^ cm^3^(STP) cm/(cm^2^ s cm Hg)). The following gases were studied: H_2_, O_2_, N_2_, CH_4_, and CO_2_. All gases were used as received from Messer Technogas s.r.o. (Czech Republic) with a purity of 99.99 %. The ideal selectivity *α*_*i/j*_ of two gases *i* and *j* was determined by the ratio (Eq. 2) [42]:

2 $$ {\alpha}_{i/j}={P}_i/{P}_j. $$

The accuracy of the measurement is given by the sum of the relative accuracies of each measured term of Eq. 1. The relative error of Δ*p*_p_/Δ*t* measured with MKS Baratron is smaller than 0.3 % plus the inaccuracy attributed to the resolution of the pressure transducer which is 1/10 of mbar. The relative standard deviation of the calibrated volume is less than 0.1 %, of the membrane area less than 0.5 %, and of the feed pressure 0.2 %. In case of the thickness of the membrane, the value can be measured as precise as 1 μm.

Sorption studies were performed on the gravimetric sorption balance IGA-002, Hiden Isochema, UK, according to the procedure described in [30]. The sorption isotherms were measured by stepwise pressure changes (pressure increase rate 100 mbar/min) within the pressure range of 0.01–4 bar. The sorption balance consists of a large capacity microbalance (5 g) with a resolution of 0.1 μg and excellent long-term stability of ±1 μg.

## Results and Discussion

**Fig. 1:**
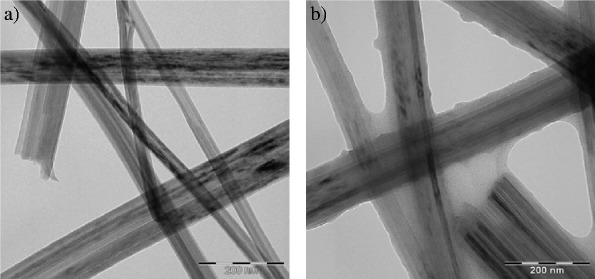
TEM images of TiNTs (**a**) and PDA-TiNTs (**b**)

The dried non-modified and modified nanotubes were studied via TEM (Fig. 1). The picture of TiNT (Fig. 1a) demonstrates the formation of long, closed, and almost aggregation-free nanotubes. After modification the nanotubes are completely covered with a thin layer of PDA as it can be seen in Fig. 1b. The obtained nanotubes posses an outer diameter of around 8–12 nm and their length varies from 100 nm to 1 μm. The thickness of the created PDA layer is about 10–12 nm.

From the measurement of the specific surface area, it was found that the *S*_BET_ of TiNT reduces significantly from 540 to 38 m^2^/g through modification with PDA (Table 1). The change in the specific surface area can be attributed to the creation of a thin polymer film on the surface of TiNTs and to the aggregation of the nanotubes.

**Table 1:** Results of *S*_BET_ determination

Sample	*S*_BET_ (m^2^/g)
TiNT	540.2
PDA-TiNT	38.4

**Fig. 2:**
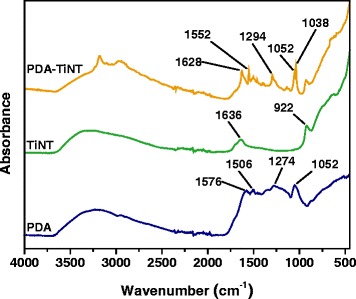
Infrared spectra of PDA, TiNT, and PDA-TiNT

The structure and chemical interactions of PDA-TiNT were examined by ATR FTIR spectroscopy, in the mid-range between 4000 and 400 cm^−1^. The IR spectra of nanotubes before and after oxidative polymerisation with PDA are shown in Fig. 2. Based on the literature [43], the broad band in the region of 3660–2350 cm^−1^ can be assigned to O–H stretching vibrations indicating the presence of hydroxyl groups on the nanotube surface. This region also corresponds to vibrations of adsorbed water molecules what confirms the small peak at around 1636 cm^−1^ (H–O–H bending vibrations). The IR spectrum of TiNT exhibits a band located at around 922 cm^−1^ which might be attributed to the Ti–O stretching vibrations involving non-bridging oxygen. The broad region around 800–400 cm^−1^ corresponds to the Ti–O–Ti vibrations in the nanotube skeleton.

In the IR spectrum of aromatic PDA a broad region between 3680 and 2350 cm^−1^ can be distinguished which can be ascribed to N–H stretching (3500–3200 cm^−1^), C–H stretching (3100–3000 cm^−1^), and H-bonded O–H stretching (3570–3200 cm^−1^). Moreover, the absorption peaks in the 1650–1430 cm^−1^ range correspond to C=C stretching in the benzene ring; however, they are partially overlapped with the ring stretching region of heterocycles containing N–H groups (1600–1300 cm^−1^). Similarly, the C–N stretching bands of aromatic amines appear in the region 1360–1250 cm^−1^ and overlap the peak attributed to the O–H bending in phenols (1410–1310 cm^−1^). The peak at 1052 cm^−1^ might correspond to the in-plane C–H bending in aromatic ring [43, 44].

The functionalisation of titanium nanotubes by PDA polymer led to visible changes in IR spectra what might be due to H-bonding interactions between both components (Fig. 2). As a first confirmation, we can point out the broadening of the band in 3680–1810 cm^−1^ region. Accordingly, in the region of lower wavelengths new peaks occur (1628, 1038 cm^-1^) and some peaks become narrower and shifted to higher wavelengths (e.g. 1552, 1294 cm^−1^) compared to the spectra of PDA. It is assumed that those changes are a result of hydrogen bond interactions between N–H and O–H groups of the PDA polymer and the Ti-O groups of the nanotubes. Nevertheless, the exact interpretation of the peaks is not possible due to measurement difficulties.

**Fig. 3:**
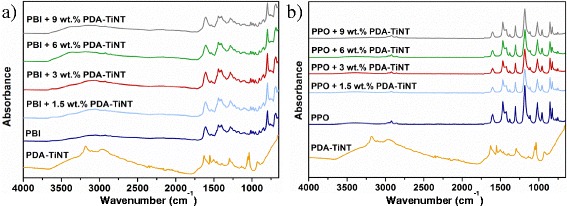
Infrared spectra of PDA-TiNT (**a**, **b**), PBI, and PBI-PDA-TiNT MMMs (**a**) as well as PPO and PPO-PDA-TiNT MMMs (**b**) with various amount of TiNT

The incorporation of PDA-TiNTs into the PBI matrix caused broadening of the peak in the region between 3680–2350 cm^−1^ (Fig. 3a). The width of the peak at 1609 cm^−1^, corresponding to C=N stretching, changed also slightly. The process was more intensive for samples with higher content of nanotubes and might signify the H-bonding interactions between N–H, O–H, Ti–O, and C=N groups present in the system.

In the case of PPO-PDA-TiNT *MMMs* (Fig. 3b) no changes of any peak has been observed, although PDA possess –OH groups which are suitable to interact with the oxygen atom present in PPO. However, the aromatic rings of PDA are very bulky, wherefore it is assumed that the interactions between the PDA-TiNT and PPO are restricted because of steric hindrance.

**Fig. 4:**
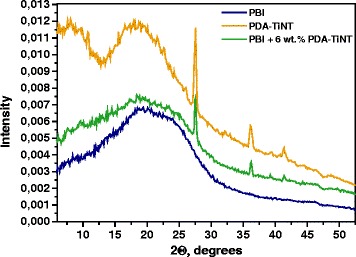
WAXS diffractogram of PDA-TiNT, PBI, and PBI with 6 wt.% of PDA-TiNT

The influence of nanotubes on the polymer matrix was studied by WAXS. Figure 4 shows the WAXS pattern for modified nanotubes, PBI, and PBI-PDA-TiNT MMM with a loading of 6 wt.% of PDA-TiNTs. As can be seen from Fig. 4, the WAXS pattern obtained from PDA-TiNT illustrates two amorphous peaks at 2Ɵ = 8° and 18°, respectively, and three crystalline peaks at 2Ɵ = 27°, 36°, and 41°. In case of pure PBI, two broad amorphous peaks were detected at 2Ɵ = 20° and 25°. With the addition of PDA-TiNT into PBI matrix, the PBI-PDA-TiNT MMM still remains its amorphous structure. Besides, some additional crystalline peaks could be detected, which can be assigned to PDA-TiNTs present in the PBI matrix.

DSC analysis was used to measure the *T*_g_ of PBI-PDA-TiNT and PPO-PDA-TiNT MMMs. This method has been applied in several studies to investigate the influence of fillers in nanocomposites on the *T*_g_. Changes in *T*_g_ were mostly ascribed to changes in chain mobility [9, 20], but effective links between the polymeric chains and the nanotube surface might be as well of relevance [6, 10]. Table 2 *depicts* the values of *T*_g_ of PBI-PDA-TiNT MMMs and PPO-PDA-TiNT MMMs, respectively, obtained from DSC measurements.

**Table 2:** *T*_g_ of neat polymers and MMMs

Sample	Amount of PDA-TiNT in membrane	*T*_g_ (°C)
PBI	0 wt.%	413.5
PBI-PDA-TiNT	6 wt.%	424.6
PPO	0 wt.%	214.6
PPO-PDA-TiNT	6 wt.%	214.4

The *T*_g_ for PBI was found to be 413.5 °C which is similar to published data [45].With addition of 6 wt% of PDA-TiNT the *T*_g_ noticeably increased. It is assumed, that the aromatic rings of PDA, which are attached on the surface of TiNT, reduces the segmental mobility of PBI polymer chains. In addition, the PDA-TiNTs seem to be able to interact with neat PBI as FTIR studies showed.

PPO-PDA-TiNT MMMs were characterised by no significant change in *T*_g_ compared to neat PPO. It is assumed that the slight differences arouse from changes in chain mobility, but not from interaction between the functional groups of PDA-TiNT with PPO which is in accordance with the FTIR results.

**Fig. 5:**
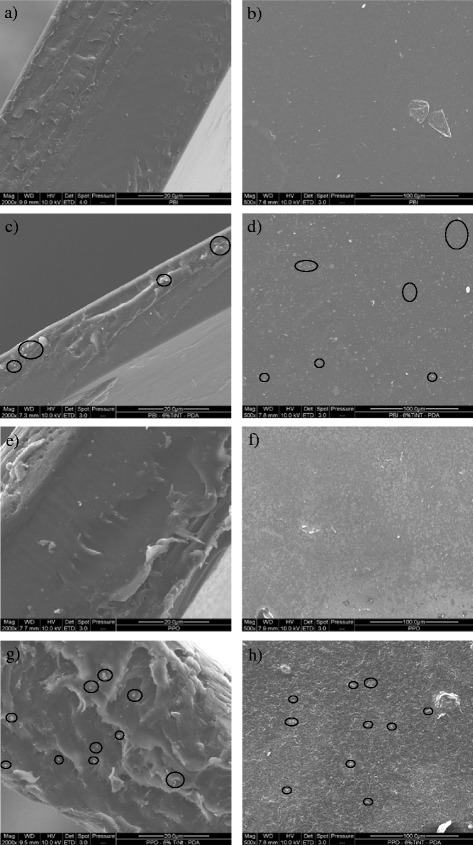
Cross sections and surface image of PBI (**a**, **b**), PBI-PDA-TiNT 6 wt.% (**c**, **d**), PPO (**e**, **f**), and PPO-PDA-TiNT 6 wt.% (**g**, **h**)

For fabricating MMMs, the fillers should disperse well in the polymer matrix. Therefore, TiNT was functionalised with PDA in order to enhance the TiNT dispergation and adhesion with the polymer matrix. The distribution of PDA-TiNT in the polymer matrix was studied by SEM. Figure 5 presents the SEM morphology of the cross section and surface of neat PBI and PPO (Fig. 5a, b; e, f), respectively, as well of PBI-PDA-TiNT and PPO-PDA-TiNT with 6 wt.% of PDA-TiNT (Fig. 5c, d; g, h). It was observed that the membranes are without undesired cracks or pin-holes. When PDA-TiNTs were incorporated into PPO, the resulting MMMs exhibited rougher surface in comparison to the neat polymer and PBI-PDA-TiNT MMMs, respectively. As the surface micrographs of the MMMs depicts, the functionalised PDA-TiNTs tend to disperse quite well in the PBI and PPO matrix independently on the PDA-TiNT content. However, it seems that the nanotubes form agglomerates, as can be seen on the surface and in the cross section of both MMMs, which might arise from inter-molecular forces and physical entanglements between the modified TiNTs. Nevertheless, it seems that the nanotubes have a good adhesion to the polymer matrix, because there is no evidence of interfacial voids in the prepared MMMs. Also, the results of the permeability measurements suggest the absence of interfacial voids as described later in this paper.

**Fig. 6:**
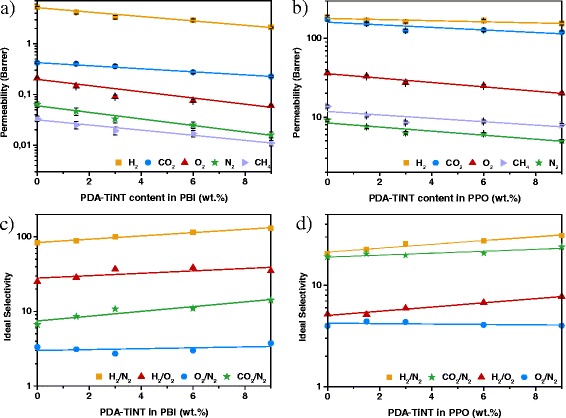
Permeability and ideal selectivity of PBI-PDA-TiNT (**a**, **c**) and PPO-PDA-TiNT MMMs (**b**, **d**)

Determination of gas permeability is a useful method for evaluating membrane performance and can also provide the information about the filler-polymer matrix compatibility. Gas separation performance of a membrane can be determined by the membrane permeability (Fig. 6a, b) and selectivity (Fig. 6c, d). In this study, both types of MMMs were tested for five different gases in order to investigate the effect of PDA-TiNT on membrane separation performance. Figure 6a, b shows the gas permeability coefficients of the PBI-PDA-TiNT MMMs (Fig. 6a) and PPO-PDA-TiNT MMMs (Fig. 6b) as a function of PDA-TiNT weight concentration. In general, for all membranes, permeability coefficients decreased in the order H_2_ > CO_2_ > O_2_ > N_2_ > CH_4_ indicating that the separation mechanism is based on solution-diffusion mechanism which is typical for most glassy polymers [46]. With increasing amount of nanotubes in PBI-PDA-TiNT MMMs and PPO-PDA-TiNT MMMs, respectively, permeability coefficients tend to decrease.

Dependent on the material used, separation properties are different. As anticipated, PBI is less permeable than PPO, because of its rigid aromatic molecular structure and its relatively high chain packing density [15, 31]. High permeability of PPO among aromatic polymeric membranes can be attributed to the ether linkages, which introduce more flexibility to the polymer chain, steric hindrance of the methyl groups and lower packing density due to the absence of polar groups [47, 48].

In both matrices, the presence of filler causes a decrease in permeability. With the increase of the PDA-TiNT amount the permeability drops. As generally known, the presence of fillers brings a sort of physical barriers in the membrane that act as obstacles in the diffusive path of a gas molecule permeating across the membrane. These obstacles increase the tortuosity for gas molecules in the present MMMs, thus decrease its permeability [5, 34].

Besides, the orientation of nanotubes inside the matrix plays also a role in the separation characteristic. It was reported in the literature that MMMs with well oriented, open-ended CNTs, which are accessible for gas molecules, exhibit increased permeability [23, 25, 49]. In the present study, as SEM results show, PDA-TiNTs are randomly orientated, sometimes interconnected or even agglomerated. Therefore, it is concluded that the accessibility of the tunnels for gas molecules is limited, and PDA-TiNTs act more as a barrier which results in permeability decrease.

Another important aspect is the influence of modifier on the membrane performance. From the obtained permeability and SEM analysis, it can be concluded that both polymers adhere well to PDA-TiNTs, although FTIR studies could detect only changes in the FTIR spectra of PBI upon addition of PDA-TiNTs. Therefore, we can conclude that the sufficient adhesion between PPO and PDA-TiNT might be caused by van der Waals forces [50]. In case of weaker interactions, gas accessible voids would be formed at the interface of PDA-TiNT and PPO, which in turn would result in a major boost of gas permeability, whereas selectivity would remain the same or would be close to the one of neat polymer [8, 51, 52].

Even though modifications led to improved adhesion for both polymers, it is interesting to note that permeability and selectivity values of PBI-PDA-TiNT MMMs are lower in comparison to those of PBI membranes with non-modified TiNTs. This result suggests that the gas transport properties of PDA have to be taken into account. When one compares the results of PPO-TiNT MMMs from earlier work [30] with the results of this study, a decrease in permeability can be observed for the PPO-PDA-TiNT MMMs along with an increase in selectivity. In contrary, PPO-TiNT MMMs showed an increase in permeability while selectivity remained almost constant. The observed increase can be explained by formed voids between the non-modified TiNTs and the PPO, which have negligible resistance to the flow of gas and thus cause an increase in permeability. The voids, however, are poorly selective, wherefore the selectivity remains constant. Thus, functionalisation of TiNTs improves the adhesion between the two phases and consequently minimise undesirable voids, which results in a decline in permeability.

The dependence of ideal selectivity of selected gas pairs on the concentration of PDA-TiNT in PBI (Fig. 6c) and PPO (Fig. 6d), respectively, is presented in Fig. 6. As the plots indicate PDA-TiNTs influence mainly the gas selectivity of PBI based MMMs. Addition of 9 wt.% PDA-TiNT to PBI resulted in an increase of 112 and 63 % in the selectivity of CO_2_/N_2_ or CO_2_/CH_4_, respectively. Similarly, the selectivity of H_2_/N_2_, H_2_/O_2_, and O_2_/N_2_ increased by 57, 40, and 12.50 %, respectively. The high selectivity values for CO_2_/N_2_ and CO_2_/CH_4_ can be explained by the polar functional groups of PDA on the TiNTs, confirmed by FTIR. Those polar groups usually exhibit a stronger interaction with polar gases, such as CO_2_, than with nonpolar gases, e.g. N_2_ or CH_4_. Thus, the polar gas solubility can be enhanced and the gas permeability can be increased which facilitates the improvement of the total CO_2_/N_2_ or CO_2_/CH_4_ selectivity [12]. Ideal selectivities of the other gas pairs further demonstrate that gases with a smaller kinetic diameter, e.g. H_2_ permeate easily through the intimately connected PBI matrix with PDA-TiNT than bigger gas molecules, e.g. N_2_ or O_2_, resulting in higher selectivity ratio.

As for PPO-PDA-TiNT MMMs, gas selectivities increase for all gas pairs besides O_2_/N_2_, which remains nearly constant, meaning that the permeabilities of O_2_ and N_2_ decreased at the same rate. Analogically, selectivity increase for the gas pairs H_2_/N_2_ and H_2_/O_2_ are similar (50 %; 49 %). The CO_2_ over N_2_ selectivity enhancement (25 %) was larger than over CH_4_ (17 %), even though the kinetic diameter of CH_4_ (3.8 Å) is greater than the one of N_2_ (3.64 Å). However, CH_4_ solubilise better in PPO than N_2_, wherefore CH_4_ preferably permeates through the MMM than N_2_, which is in accordance with the literature [53–55].

Comparing the results of PBI-PDA-TiNT and PPO-PDA-TiNT MMMs, one can see that the addition of PDA-TiNT influence the separation performance of PBI-PDA-TiNT MMMs more significant than of PPO-PDA-TiNT MMMs.

**Fig. 7:**
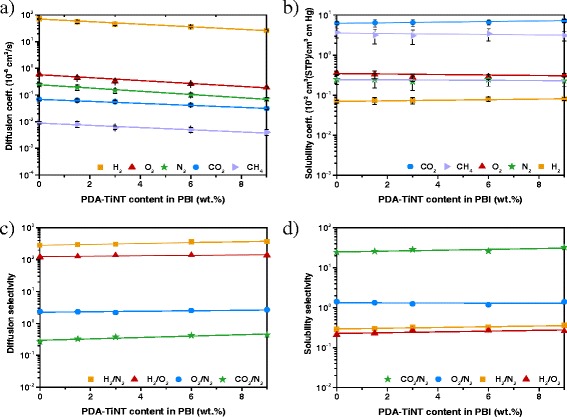
Diffusion coefficients (**a**) and solubility coefficients (**b**) with the corresponding selectivities (**c**, **d**) of PBI-PDA-TiNT MMMs (**b**, **d**)

**Fig. 8:**
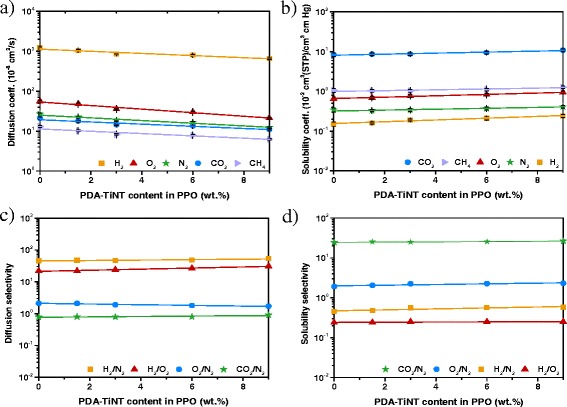
Diffusion coefficients (**a**) and solubility coefficients (**b**) with the corresponding selectivities (**c**, **d**) of PPO-PDA-TiNT MMMs (**b**, **d**)

The differences in permeabilities of each MMMs can be better understood by analysing the diffusion and solubility coefficients, since the permeability *P* of a gas is proportional to the diffusivity *D* and solubility *S* of a gas in the membrane (*P* = *D* × *S*) [42]. Moreover, for a given polymeric membrane, *D* mainly depends on the kinetic diameter of a gas molecule and *S* mainly on the condensability of a gas molecule [20]. Upon adding inorganic nanofillers, gas transport properties may be affected in the following ways: (a) the incorporation of nonporous or impermeable fillers in a polymer membrane will lead to a reduction of permeability due to increased tortuosity of the diffusion path as well as reduced solubility of the separating gas molecules in the polymer matrix [34], (b) the interactions between the nanoparticles and the polymer chains or penetrants, respectively, are strong and significantly change the diffusivity and solubility of penetrants [25], (c) the nanoparticles affects the polymer chain stiffness or mobility and therefore the gas diffusion by increasing or decreasing of the free volume of the polymer chains [34], (d) the interaction between polymer-chain segments and nanoparticles may increase or decrease the formation of voids (defects between polymer/nanoparticle interface), and therefore deteriorates the gas diffusion [56], (e) the modification of nanoparticles introduces functional groups on the surface of the nanoparticles, which may interact with more condensable gases such as CO_2_ and therefore improve the gas’ solubility in the MMMs [57]. Figure 7 shows the diffusion and solubility coefficients with the respective selectivities of neat PBI and PBI-PDA-TiNT MMMs. The values for neat PPO and PPO-PDA-TiNT MMMs are presented in Fig. 8.

As can be seen from Figs. 7a and 8a, gas diffusivities of all studied gases decreased with PDA-TiNT loading. After incorporation of 9 wt.% PDA-TiNTs to PBI, diffusion coefficients for H_2_, O_2_, N_2_, CO_2_, and CH_4_ decreased by around 37, 32, 28, 46, and 39 %, respectively (Fig. 7c). In case of PPO, the diffusion coefficients decreased about 54, 38, 46, 52, and 48 % for H_2_, O_2_, N_2_, CO_2_, and CH_4_, respectively (Fig. 8c). These results suggest that there are no voids between PDA-TiNTs and polymer chains leading to higher gas diffusion resistance and therefore to decreased diffusion coefficients. If there had been some voids, gas separation performance would be affected to a great extent, because the gaps at the interface between the nanotubes and the polymer provide a less resistive route for gases; thus, gas permeability would increase. Moreover, it is assumed that the addition of PDA-TiNTs facilitates polymer chain packing and reduces the free volume between the polymer chains due to the decreased diffusion coefficients. In addition, it seems, that the channels of the added nanotubes are not readily accessible for the gas molecules, elsewise the prepared MMMs would possess higher gas permeabilities due to a more effectively transport of the gas molecules through the tunnels of the nanotubes.

Despite the decrease in diffusivity, the solubility of PBI-PDA-TiNT MMMs remained relatively unchanged for H_2_, N_2_, O_2_, and CH_4_ with the increase of nanotubes, besides for CO_2_ (Fig. 7b). CO_2_ is a more condensable gas than all the other studied gases. The increased gas condensability leads to the enhancement of the solubility of the gas in the polymer matrix, which confirms the dominancy of the solution mechanism in the permeation of CO_2_ through the MMMs upon addition of PDA-TiNTs. Moreover, the strong polar affinity between the functional groups of PDA on the surface of the nanotubes and CO_2_ results in an increased CO_2_ solubility. With the increase of PDA-TiNT in the matrix the CO_2_ solubility increases due to the larger content of CO_2_-facilitated transport sites in the membranes, which leads to a steady increase of CO_2_/N_2_ solubility selectivity (Fig. 7d). In contrary, the solubility values of the PPO-PDA-TiNT MMMs showed for all gases an increase in solubility coefficients (Fig. 8b). Highest increase of solubility selectivity was found for the gas pair H_2_/N_2_, which might be due to the differences in kinetic diameters of these gas molecules. If one compares the solubility values of the neat polymers, it can be found that the solubility coefficients (Fig. 8d) of all gases besides CH_4_ are higher for PPO than for PBI (Fig. 7d); thus, the gases can solubilise better in PPO than in PBI. The differences in the solubilities between those two polymers are caused by the different molecular structures of PBI and PPO.

From the obtained diffusivity and solubility results (Figs. 7, and 8), it can be concluded that the addition of PDA-modified TiNTs to PBI or PPO deteriorates the permeability properties by decreasing gas diffusivity of all studied gases due to enhanced chain packing density and reduced chain segment mobility. In addition, the incorporation of modified nanotubes into PBI or PPO enhances the solubility of condensable gases by increasing the number of functional groups. Moreover, it is believed that the modified TiNTs act as impermeable filler which are lowering the permeability of all gases; hence, hindering the diffusion of the gases through the MMMs. Furthermore, the MMMs have no evidence of unselective voids.

**Fig. 9:**
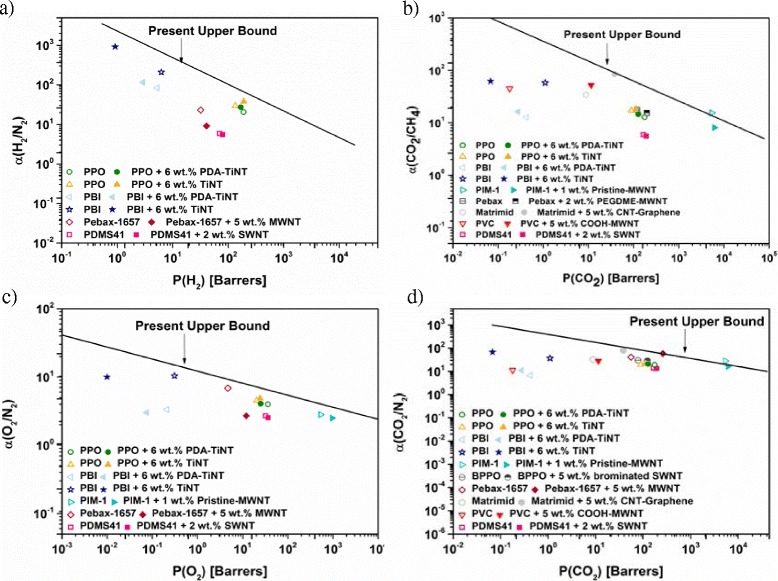
Robeson diagrams of the gas pairs: **a** H_2_/N_2_, **b** CO_2_/CH_4_, **c** O_2_/N_2_, and **d** CO_2_/N_2_

To clearly display the membrane performances, some of the pure gas data have been encompassed in the Robeson plot (Fig. 9). This plot represents the limits of the selectivity-permeability behaviour of neat polymers via the upper bound [14, 58]. Well above the upper bound is the commercially attractive region for membrane preparation [52]. Most inorganic membranes are situated there. However, these materials are expensive and difficult to prepare, wherefore the combination of polymers with fillers seems to be a promising solution to exceed this upper bound [8]. Figure 9 *depicts* the existing literature data of selected MMMs based on nanotubes [12, 20–25, 30] and the data of selected membranes of the present work in order show the impact of nanotubes on different types of polymers and to show the upper bound limits for the gas pairs H_2_/N_2_, CO_2_/CH_4_, O_2_/N_2_, and CO_2_/N_2_.

As can be seen from Fig. 9 the composition of MMMs plays an important role. Depending on the choice of material and filler, the obtained results fall close to the trade-off line. The addition of different types of nanotubes caused either an increase in permeability and decrease in selectivity or vice versa. Only in the case of Matrimid-based MMMs, an increase in both parameters could be observed. Nevertheless, additional studies on MMMs need to be performed in order to overcome this upper bound.

Table 3 presents additionally the permeability and selectivity data of various types of PPO and PBI MMMs prepared in this work [30–34] and the ones reported elsewhere.

**Table 3:** Technical achievable performance according to types of membranes investigated by different research groups

Polymer	Filler (wt.%)	Permeability (Barrer)					Selectivity				Reference
		*P*_H2_	*P*_O2_	*P*_N2_	*P*_CH4_	*P*_CO2_	*α*_CO2/N2_	*α*_CO2/CH4_	*α*_H2/N2_	*α*_H2/CO2_	
PPO	SBA-15^a^										[32]
	0	134.05	–	3.80	4.70	69.20	17.9	14.8	37.1	2.1	
	10	92.82	–	2.10	1.51	53.20	26.8	36.0	49.7	1.8	
	Silica^a^										[33]
	0	82.17	–	–	–	48.81	–	–	–	1.68	
	10	548.7	–	–	–	154.05	–	–	–	3.56	
	TINT^a^										[30]
	0	134.255	20.326	4.508	5.203	88.7	19.68	17.05	29.78	1.51	
	6	190.532	24.379	5.035	6.135	112.298	22.3	18.3	37.84	1.7	
	TINT^b^										This work
	0	188.88	36.41	9.14	13.716	175.04	19.15	12.76	20.66	1.08	
	6	168.68	25.00	6.14	8.8	127.78	20.81	14.52	27.48	6.75	
PBI	ZIF-8^a^										[31]
	0	4.1	–	–	–	0.46	–	–	–	8.9	
	30	82.5	–	–	–	6.9	–	–	–	12.0	
	Silica^a^										[34]
	0	–	–	0.007	0.005	0.025	3.5	4.96	–	–	
	20	–	–	0.002	0.010	0.11	71.3	11.4	–	–	
	TINT^a^										[30]
	0	6.268	0.312	0.030	0.019	1.101	36.7	57.95	208.93	5.693	
	6	0.920	0.010	0.001	0.001	0.068	68	68	920	13.53	
	TINT^b^										This work
	0	5.24	0.21	0.063	0.033	0.421	6.68	12.64	83.17	12.45	
	6 wt.%	2.90	0.075	0.025	0.017	0.275	11.00	16.18	116	10.55	

^a^Non-modified

^b^Modified

Table 3 reveals that PPO and PBI have been utilised in several types of research as a polymer matrix for membrane preparation. The gas transport properties of the neat membranes are in accordance with those of other works. Upon addition of any kind of filler, different effects on the separation performance can be observed. In general, the addition of silica, ZIF-8, SBA-15, and non-modified or modified TiNTs, respectively, to PPO or PBI cause an increase in gas selectivities. As far as permeabilities are concerned, they either increased or decreased. This can be explained by good adhesion of the polymer chains on the surface of the filler. Poor adhesion of the polymer onto the filler can cause voids between the interface of polymer chains and fillers, which leads to an increase in permeability, whereas selectivity remains almost the same. In conclusion, the employment of fillers can result in high selective membranes with superb permeation rates depending on the choice of polymer and filler.

**Fig. 10:**
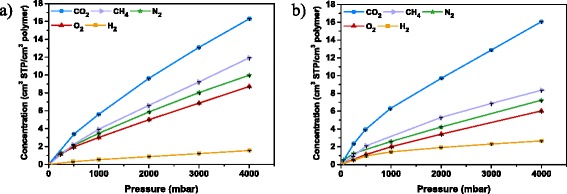
Sorption isotherms of PBI (**a**) and 6 wt.% PBI-PDA-TiNT MMM (**b**)

Figure 10 presents the sorption isotherms of neat PBI membrane and 6 wt.% PBI-PDA-TiNT MMM, respectively, for the gases H_2_, O_2_, N_2_, CH_4_, and CO_2._

For describing the sorption behaviour of glassy polymers, the so-called dual-mode sorption model is often used (Eq. 3) [46, 59–61].

3 $$ \begin{array}{l}C={\mathrm{k}}_{\mathrm{D}}p\kern0.5em +\kern0.5em \frac{{\mathrm{C}}_{\mathrm{H}}^{\prime } bp}{1+ bp},\\ {}\underset{\begin{array}{l}\mathrm{Henry}\hbox{'}\mathrm{s}\\ {}\mathrm{law}\ \mathrm{term}\end{array}}{\underbrace{\kern2em }}\kern1em \underset{\begin{array}{l}\mathrm{Langmuir}\\ {}\mathrm{term}\end{array}}{\underbrace{\kern2em }}\end{array} $$

where *C* is the total gas concentration in a glassy polymer, k_D_ is the Henry’s law parameter, C^’^_H_ is the Langmuir sorption capacity, and b is the Langmuir affinity parameter.

At low to moderate pressures, the sorption is determined by the Langmuir term and describes the non-equilibrium region of the curve. Furthermore, it characterises the sorption on the holes or “microvoids” from the non-equilibrium nature of glassy polymers. At higher pressures, Henry’s law solubility predominates. Its corresponding Henry’s law term describes the equilibrium region and is related to the dissolution of gases into the dense equilibrium structure of rubbery polymers.

In general, the measured sorption data for the more condensable gases CO_2_ and CH_4_ can be well fitted by the dual-mode sorption model (Fig. 10). Even the lower condensable gases N_2_ and O_2_ exhibit slightly nonlinear pressure dependence. In case of H_2_, which does not exhibit significant gas sorption, follows the Henry’s type gas sorption model, which demonstrates a linear relationship of gas sorption with pressure.

For PBI, sorption isotherms are similar to previously reported ones [30]. In contrary, the 6 wt.% PBI-PDA-TiNT MMM (Fig. 10) exhibits a significant decrease in gas sorption capacity of CH_4_, N_2_, O_2_, and H_2_. In previous study with non-modified TiNTs, it was found that the addition of fillers increase the sorption capacity [30], which is in agreement with other published data of MMMs made of nanotubes or other nanoparticles, respectively [56, 62, 63]. In the present case, functionalisation of TiNTs alters the lattice structure of TiNTs which led to a remarkable decrease in the specific area as the values of Table 1 show. This might cause a decline in the sorption capacity of the surface or inner side of TiNTs. Besides, functionalisation improves the filler-polymer interface compatibility for MMMs as can be concluded from IR results, wherefore the gas molecules cannot adsorb in the interlayer spacing between polymer and nanotubes. These major changes achieved in the material’s structure lower considerably its sorption capacity for the gases CH_4_, N_2_, O_2_, and H_2_.

In case of CO_2_, the sorption capacity of the 6 wt.% PBI-PDA-TiNT MMM is in the same amount as of neat PBI membrane due to the inclusion of new sorption sites throughout the addition of modified nanotubes. The abundant CO_2_ selective groups, on the surface of TiNTs, enlarge the CO_2_ adsorption capacity due to increased polar interactions between the gas molecules and the existing functionalised surface. Similar trends of increased CO_2_ sorption capacity were also observed in other reported MMMs [12, 63] and were demonstrated in our previous study with non-modified TiNTs [30]. To conclude, the improvement of sorption capacity by PDA-TiNTs can be attributed to the increase of sorption sites.

## Conclusions

MMMs were prepared using TiNTs functionalised with PDA and a PBI and PPO, respectively, as the polymer matrix. TEM and *S*_BET_ analysis have confirmed the successful PDA functionalisation of TiNTs. The SEM images of the prepared MMM revealed that the functionalised TiNTs are well dispersed throughout the both matrices. Besides, it seems that the nanotubes form agglomerates in the membrane due to functionalisation by PDA. As DSC studies showed, with addition of 6 wt% of PDA-TiNT to PBI the *T*_g_ noticeably increased, whereas for the PPO matrix not. According to FTIR studies, PBI and PDA-TiNT interacts possibly via hydrogen bonds. In contrary, no interactions between PPO and PDA-TiNT were observed. In any case, it seems, there is good interfacial adhesion and the absence of voids between PDA-TiNTs and both polymer matrices, as the gas permeabilities decreased for all gases with the addition of PDA-TiNTs. Lowest permeabilities were observed with the highest content of PDA-TiNT in the MMMs. It is clear that addition of SWNTs to a polymer matrix can improve certain selectivities as well as permeabilities of small molecules. On the other hand, the addition of TiNTs to a polymer matrix can improve certain gas selectivities, especially for CO_2_/N_2_ and CO_2_/CH_4_. Further, the sorption capacity of neat PBI and PBI-PDA-TiNT was studied for H_2_, O_2_, N_2_, CH_4_, and CO_2_. The sorption isotherms elucidated that the presence of PDA-TiNTs in PBI has a positive effect on CO_2_ sorption.

## ᅟ

ATR: Attenuated total reflectance
BET: Brunauer-Emmett-Teller
CNT: Carbon nanotube
MCT: Mercury–cadmium–telluride
MMM: Mixed matrix membrane
MOF: Metal organic framework
MWNT: Multi-walled nanotubes
PBI: Poly(5,5-bisbenzimidazole-2,2-diyl-1,3-phenylene)
PDA: Polydopamine
PDA-TiNT: PDA-functionalised TiNT
PPO: Poly(2,6-dimethyl-1,4-phenylene oxide)
*S*_BET_: Specific surface area
TEM: Transmission electron microscope
TiNT: Titanate nanotube
WAXS: Wide-angle X-ray scattering

## Symbols

*A*: Membrane area
b: Langmuir affinity constant
*C*: Total gas concentration
c_H_: Langmuir capacity constant
k_D_: Henry’s constant
*l*: Membrane thickness
*P*: Permeability
*p*: Penetrant partial pressure
*p*_i_: Feed pressure
*p*_p_: Pressure increase
R: Ideal gas constant
*S*: Solubility coefficient
*T*: Temperature
*t*: Time
*V*_p_: Volume of the product part

## Greek Symbol

*α*: Selectivity
